# Neuroprotective Effects and Mechanisms of Procyanidins In Vitro and In Vivo

**DOI:** 10.3390/molecules26102963

**Published:** 2021-05-17

**Authors:** Juan Chen, Yixuan Chen, Yangfan Zheng, Jiawen Zhao, Huilin Yu, Jiajin Zhu, Duo Li

**Affiliations:** 1 Department of Food Science and Nutrition, Zhejiang University, Hangzhou 310000, China; CHENJUAN544676305@163.com (J.C.); cyx901109@126.com (Y.C.); zhyfan2017@163.com (Y.Z.); 21813084@zju.edu.cn (J.Z.); yuhl0323@126.com (H.Y.); duoli@qdu.edu.cn (D.L.); 2 Institute of Nutrition & Health, Qingdao University, Qingdao 266000, China

**Keywords:** procyanidins, neuroprotective, zebrafish, PC12 cells, Nrf2/ARE pathway

## Abstract

This study evaluated the neuroprotective effects and mechanisms of procyanidins (PCs). In vitro, rat pheochromocytoma cells (PC12 cells) were exposed to PCs (1, 2 or 4 μg/mL) or N-Acetyl-L-cysteine (NAC) (20 μM) for 24 h, and then incubated with 200 μM of H_2_O_2_ for 24 h. Compared with H_2_O_2_ alone, PCs significantly increased antioxidant activities (e.g., glutathione peroxidase (GSH-Px), superoxide dismutase (SOD), catalase (CAT)), decreased levels of reactive oxygen species (ROS) and malondialdehyde (MDA), and increased nuclear factor-erythroid 2-related factor 2 (Nrf2) accumulation and increased the expression of quinone oxidoreductase 1 (NQO1), heme oxygenase 1 (HO-1), glutamate-cysteine ligase modifier subunit (GCLM), and glutamate-cysteine ligase catalytic subunit (GCLC). In vivo, zebrafish larvae (AB strain) 3 days post-fertilization (dpf) were exposed to NAC (30 μM) or PCs (4, 8 or 16 μg/mL) in the absence or presence of 300 μM of H_2_O_2_ for 4 days. Compared with H_2_O_2_ alone, PCs enhanced antioxidant activities (e.g., GSH-Px, CAT, and SOD), decreased levels of ROS and MDA, and enhanced Nrf2/ antioxidant response element (ARE) activation and raised expression levels of NQO1, HO-1, GCLM, and GCLC. In conclusion, these results indicated that PCs exerted neuroprotective effects via activating the Nrf2/ARE pathway and alleviating oxidative damage.

## 1. Introduction

Neurodegenerative disease refers to the slow and gradual degeneration of neurons in the human brain, which leads to a variety of motor and/or cognitive problems and eventually leads to neuronal death [1]. With the increasing number of elderly people, the incidence rate of senile neurodegenerative diseases is increasing rapidly [2,3]. Although the underlying mechanisms of neurodegeneration are still unclear, considerable evidence shows that oxidative stress caused by overproduction reactive oxygen species (ROS) and/or impairment of the antioxidant defense system plays a key role in these diseases [4,5,6].

In the process neurodegeneration, there is an out-off-balance between the production of intracellular ROS and the ability of the antioxidant defense system to clear ROS and/or repair ROS-mediated damage [7]. Excessive production of ROS leads to macromolecular damage. Therefore, the accumulation of cellular macromolecular damage leads to necrosis [8,9]. Consequently, supplement of antioxidants to eliminate excess ROS may help to prevent neurodegenerative diseases. Natural bioactive compounds are safe with little or no side effects. Therefore, increasing attention has been paid to the discovery of antioxidant compounds from natural resources.

Procyanidins (PCs) are natural phenolic compounds of flavonoids and contain oligomers of monomers catechin and epicatechin [10,11,12]. PCs are natural nutrients and antioxidants, and their antioxidant effects are stronger than those of vitamin C and vitamin E [13]. However, the neuroprotective effects and mechanism of PCs regarding oxidative damage in vivo and in vitro remain unclear.

Zebrafish are freshwater tropical cyprinid fish confined to India. Interestingly, roughly 87% of human disease-related genes are linked to homologous genes in zebrafish [14]. Relative to experimental mouse models, utilization of zebrafish as a model system is associated with shorter experimental periods, reduced costs, and more amenable gene manipulations [15]. In terms of in vitro models, rat pheochromocytoma cells (PC12 cells) are similar to neurons in terms of morphology, structure, and function [16]. Their features are highly consistent with those of primary cultured neurons, and have experimental advantages in terms of being easily and stably maintained in culture [17]. Furthermore, PC12 cells have been widely used as in vitro models for studying nerve cells [18,19,20]. Hydrogen peroxide (H_2_O_2_) is a type of ROS that can permeate through cell membranes and initiate oxidative stress [21]. Numerous reports have demonstrated that H_2_O_2_ can be used to establish models of oxidative stress [22,23,24]. In this study, we evaluated the neuroprotective effects and mechanisms of PCs in PC12 cells and zebrafish.

## 2. Results

### 2.1. Effects of PCs on H_2_O_2_-Treated Damage of PC12 Cells

**Figure 1:**
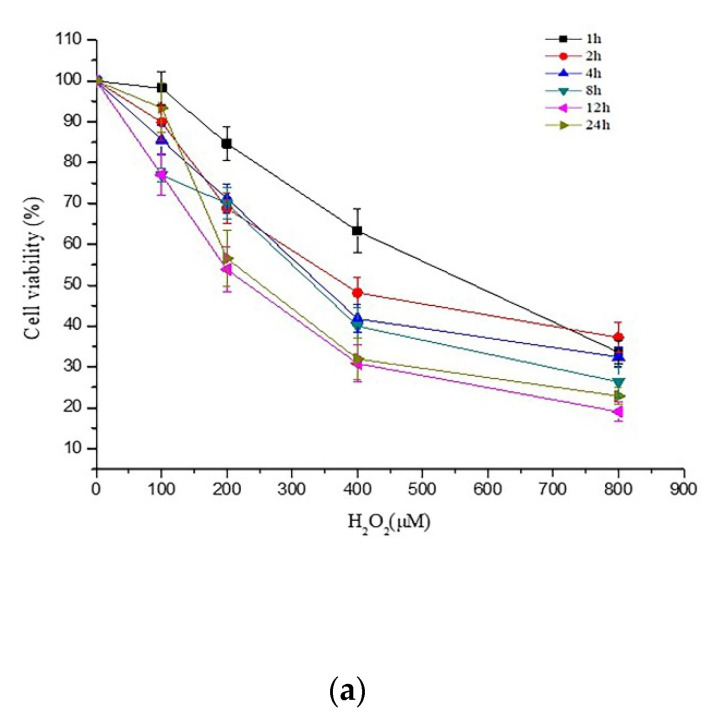

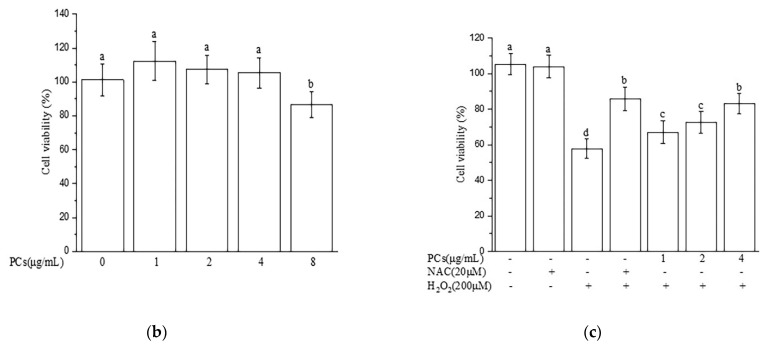
Effects of PCs on H_2_O_2_-treated cytotoxicity of PC12 cells. Cell viability was detected by CCK-8 assays. (**a**) Cytotoxic effects of H_2_O_2_ at different concentrations in PC12 cells; (**b**) cytotoxic effects of PCs at different concentrations in PC12 cells; (**c**) PCs attenuated H_2_O_2_-induced decreases in cell viability. Data are expressed as the mean ± SD. All experiments were conducted six times. Values with different letters above each bar represent significant differences (*p* < 0.05, one-way ANOVA).

PC12 cells were exposed to 200 μM H_2_O_2_ for 24 h, and the cell viability decreased to 56.5% (Figure 1a). Pretreatment of cells with PCs alone at 1, 2, or 4 μg/mL had no effect on cell viability, whereas 1, 2, and 4 μg/mL PCs treatment significantly protected PC12 cells from H_2_O_2_-treated cytotoxicity by ameliorating cell viability (Figure 1b,c, *p* < 0.05 versus H_2_O_2_-treated group). However, PCs treatment alone at 8 μg/mL decreased cell viability (Figure 1b). N-Acetyl-L-cysteine (NAC), which is known to protect against H_2_O_2_-induced oxidative damage, was used as a positive control.

### 2.2. Effects of PCs on Oxidative Stress of PC12 Cells Treated with H_2_O_2_

**Figure 2:**
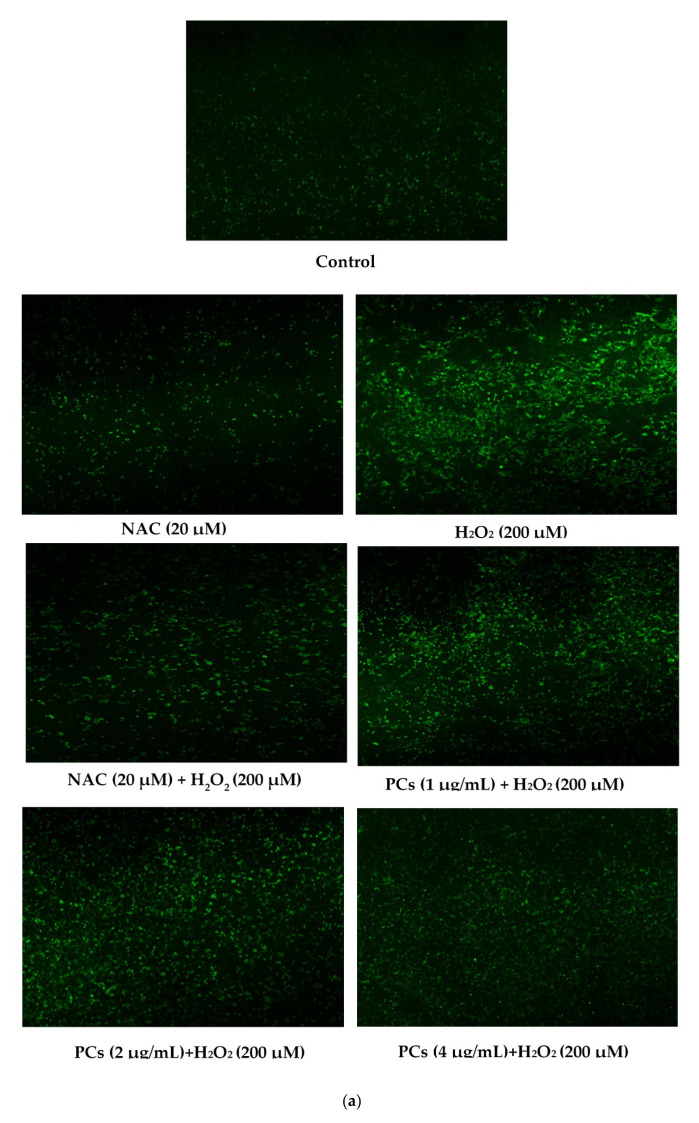

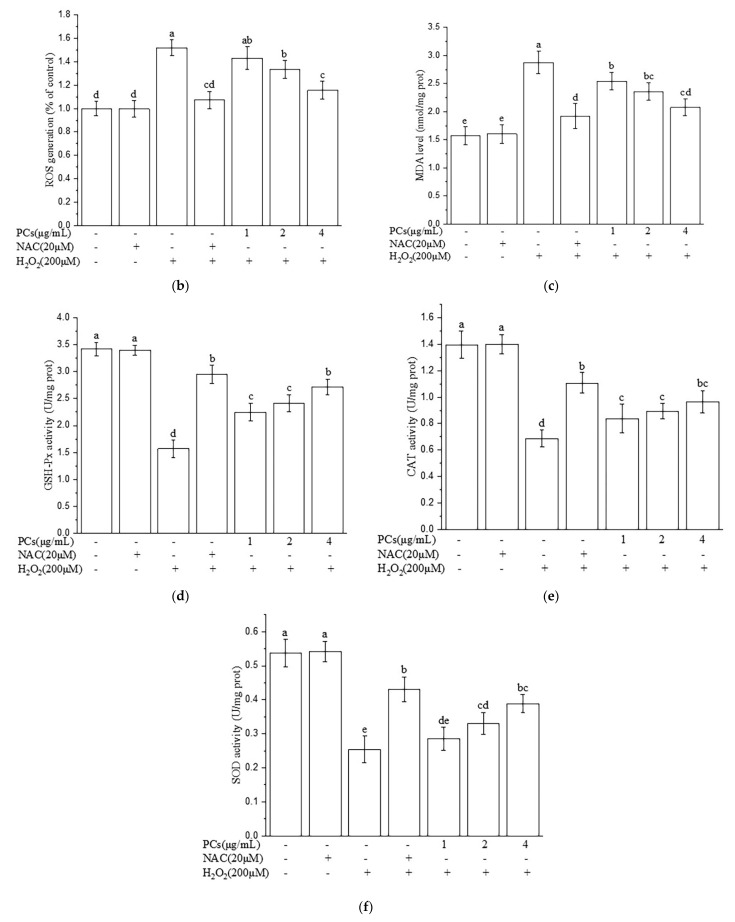
PCs attenuate H_2_O_2_-treated oxidative stress and increase antioxidant activities in PC12 cells. (**a**) Representative fluorescence photomicrographs of PC12 cells; (**b**) ROS levels were measured by software; (**c**) MDA levels; (**d**) GSH-Px activity; (**e**) CAT activity; (**f**) SOD activity. Data are expressed as the mean ± SD. All experiments were conducted three times. Values with different letters above are significantly different (*p* < 0.05, one-way ANOVA).

ROS and malondialdehyde (MDA) levels indicate the severity of oxidative damage. PC12 cells exposed to 200 μM H_2_O_2_ for 24 h raised the levels of ROS and MDA, and 2 and 4 μg/mL PCs pretreatment inhibited the levels of ROS and MDA (Figure 2a–c, *p* < 0.05 versus H_2_O_2_-induced group). To further evaluate the antioxidant effects of PCs in H_2_O_2_-induced PC12 cells, we measured glutathione peroxidase (GSH-Px), catalase (CAT), and superoxide dismutase (SOD) activities, and observed that H_2_O_2_ treatment suppressed GSH-Px, CAT, and SOD activities. However, H_2_O_2_-induced effects were rescued by pretreatment with PCs at 2 and 4 μg/mL (Figure 2d–f, *p* < 0.05 versus H_2_O_2_-treated group).

### 2.3. Effects of PCs on the Nuclear Factor-Erythroid 2-Related Factor 2 (Nrf2)/ARE Pathway in H_2_O_2_-Treated PC12 Cells

**Figure 3:**
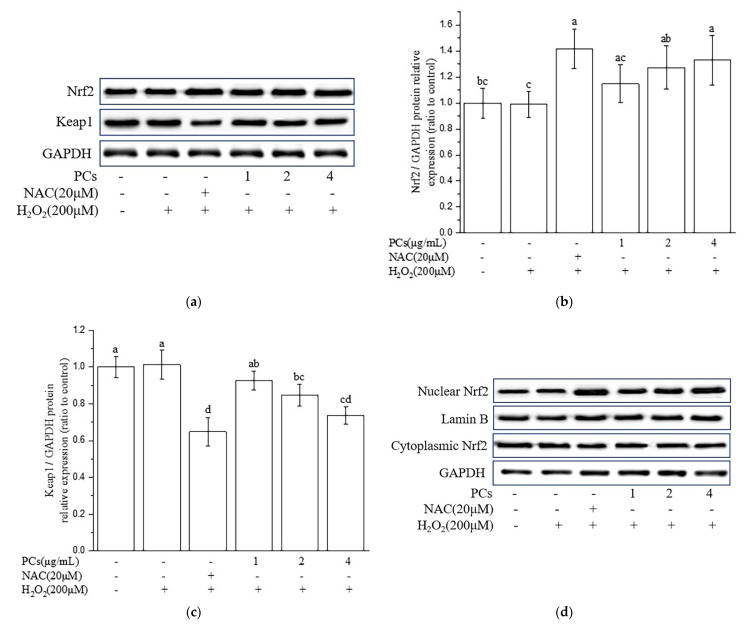

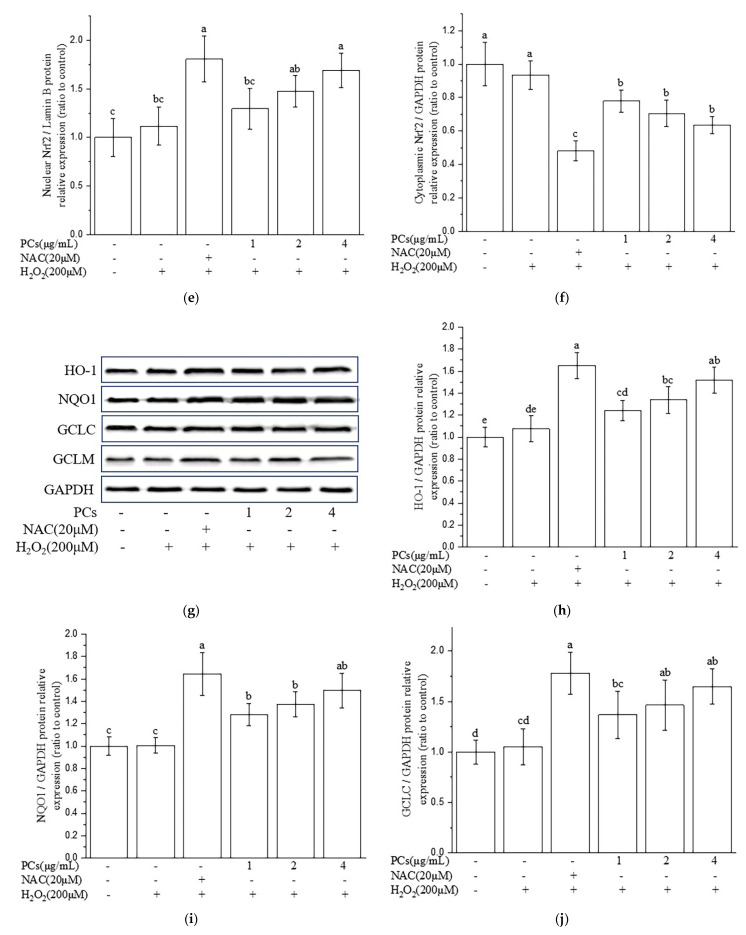

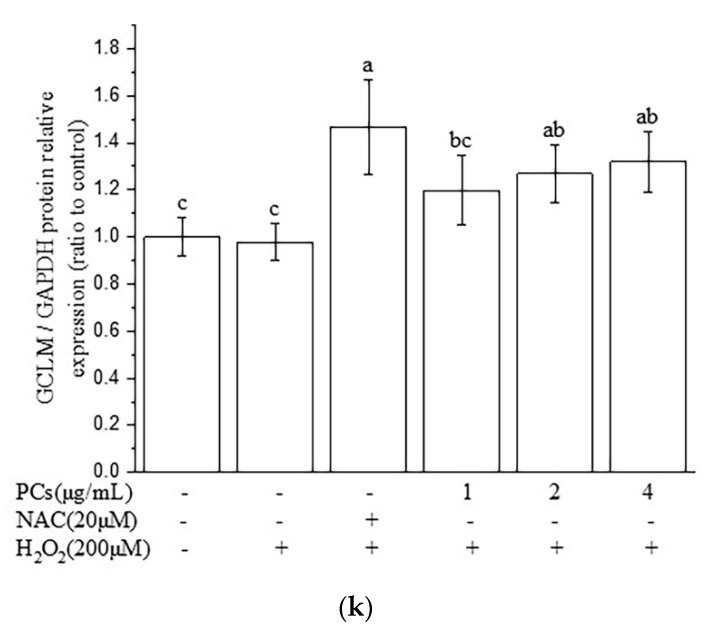
Effects of PCs on the Nrf2/ARE pathway in H_2_O_2_-treated PC12 cells. (**a**) Protein levels of Nrf2 and Keap1, as determined by Western blotting; (**b**) Nrf2/GAPDH protein relative expression (ratio to control); (**c**) Keap1/GAPDH protein relative expression (ratio to control); (**d**) protein expression levels of nuclear Nrf2 and cytoplasmic Nrf2, as determined by Western blotting; (**e**) nuclear Nrf2/LaminB protein relative expression (ratio to control); (**f**) cytoplasmic Nrf2/GAPDH protein relative expression (ratio to control); (**g**) protein levels of HO-1, NQO1, glutamate-cysteine ligase catalytic subunit (GCLC), and GCLM, as determined by Western blotting; (**h**) HO-1/GAPDH protein relative expression (ratio to control); (**i**) NQO1/GAPDH protein relative expression (ratio to control); (**j**) GCLC/GAPDH protein relative expression (ratio to control); (**k**) GCLM/GAPDH protein relative expression (ratio to control). Data are expressed as the mean ± SD. All experiments were conducted three times. Values with different letters above are significantly different (*p* < 0.05, one-way ANOVA).

We found that the level of Nrf2 was upregulated and the expression of Keap1 was downregulated following PCs (2 and 4 μg/mL) pretreatment prior to H_2_O_2_ treatment, as compared to these levels in the H_2_O_2_-induced group (Figure 3a–c, *p* < 0.05 versus H_2_O_2_-induced group). Furthermore, PCs (4 μg/mL) facilitated nuclear localization of Nrf2 under H_2_O_2_-lesioning conditions (Figure 3d–f, *p* < 0.05 versus H_2_O_2_-induced group). Additionally, Western blotting showed that heme oxygenase 1 (HO-1), quinone oxidoreductase 1 (NQO1), glutamate-cysteine ligase catalytic subunit (GCLC), and glutamate-cysteine ligase modifier subunit (GCLM) levels were upregulated following PCs (2 and 4 μg/mL) treatment compared with H_2_O_2_ alone (Figure 3g–k, *p* < 0.05 versus H_2_O_2_-induced group).

### 2.4. Nrf2/ARE Signaling Is Responsible for PCs-Mediated Antioxidative Effects

**Figure 4:**
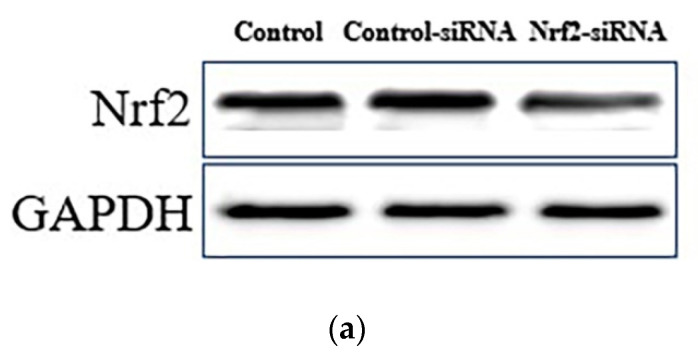

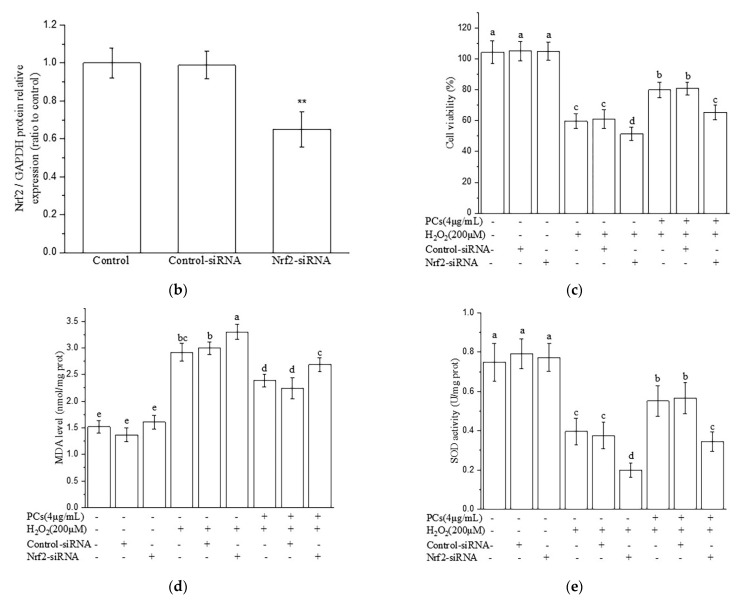
Nrf2/ARE signaling is responsible for PCs-mediated antioxidative actions in H_2_O_2_-treated PC12 cells. (**a**) Knockout efficiency was detected by determination of Nrf2 protein expression using Western blotting; (**b**) Nrf2/GAPDH protein relative expression (ratio to control); (**c**) cell viability; (**d**) MDA levels; (**e**) SOD activity. Data are expressed as the mean ±SD. All experiments were conducted three times. Values with different letters above are significantly different, (*p* < 0.05, ** *p* < 0.01 relative to control group, one-way ANOVA).

As shown in Figure 4a,b, Nrf2 siRNA inhibited the expression of Nrf2. Meanwhile, Nrf2 siRNA treatment eliminated the protective effects of PCs against H_2_O_2_-treated oxidative damage, as evidenced by the reduced cell viability in the Nrf2-siRNA-transfected group (Figure 4c). Simultaneously, the actions of PCs on MDA content and SOD activity were reversed by Nrf2 knockdown under H_2_O_2_-lesioning conditions (Figure 4d,e).

### 2.5. Effects of PCs on Motility of Zebrafish Larvae Following H_2_O_2_ Treatment

**Figure 5:**
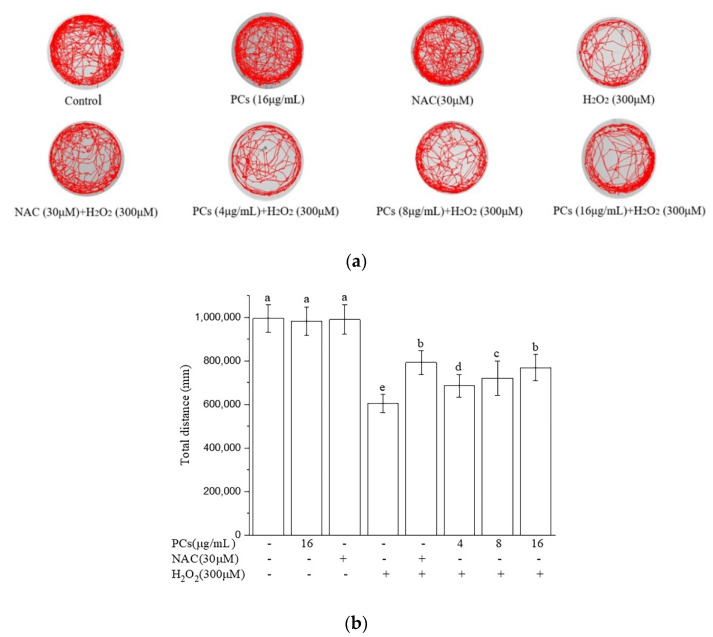
Effects of PCs on motility of H_2_O_2_-treated zebrafish larvae. (**a**) Typical patterns of swimming traces of zebrafish larvae in each group; (**b**) average total distance of zebrafish larvae in each group. Data are shown as the mean ± SD. All experiments were repeated three times. Values with different letters above are significantly different (*p* < 0.05, one-way ANOVA).

H_2_O_2_-induced oxidative damage decreased the total swimming distance of zebrafish larvae. PCs treatment (4, 8, and 16 μg/mL) rescued H_2_O_2_-induced locomotor deficits, as evidenced by an increased movement distance relative to that of the H_2_O_2_ group (Figure 5a,b, *p* < 0.05 versus H_2_O_2_-treated group). NAC, which is known to protect against H_2_O_2_-induced oxidative damage, was used as a positive control. Moreover, PCs (16 μg/mL) or NAC (30 μM) alone did not affect locomotor behavior of normal zebrafish larvae.

### 2.6. Effects of PCs on Oxidative Stress of Zebrafish Larvae Treated with H_2_O_2_

**Figure 6:**
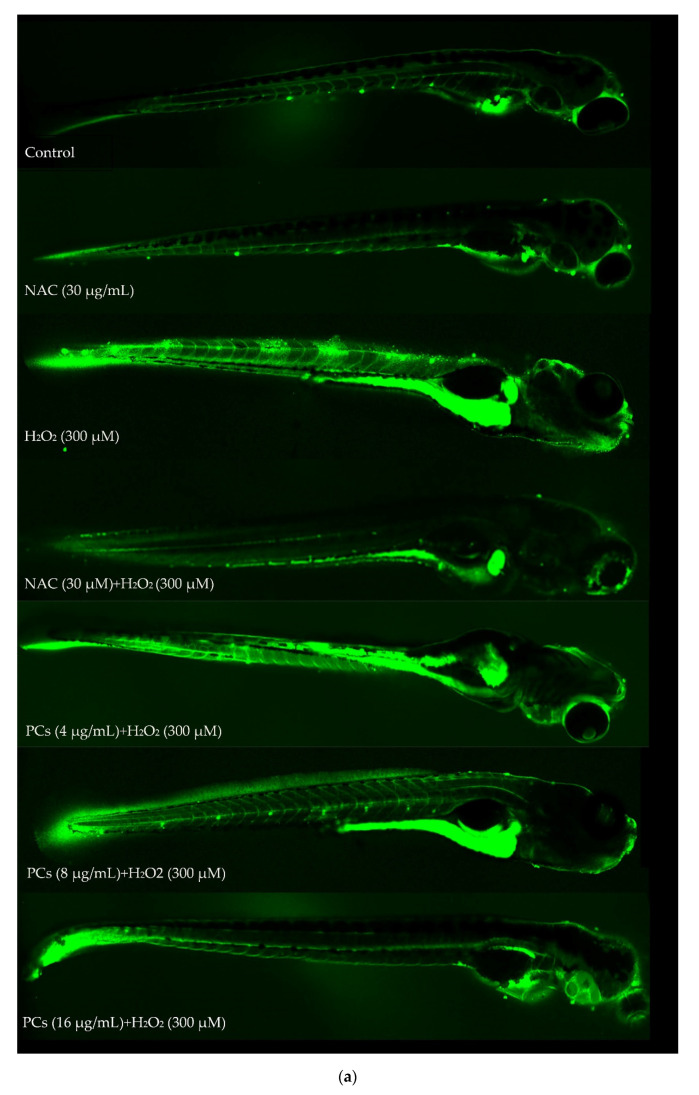

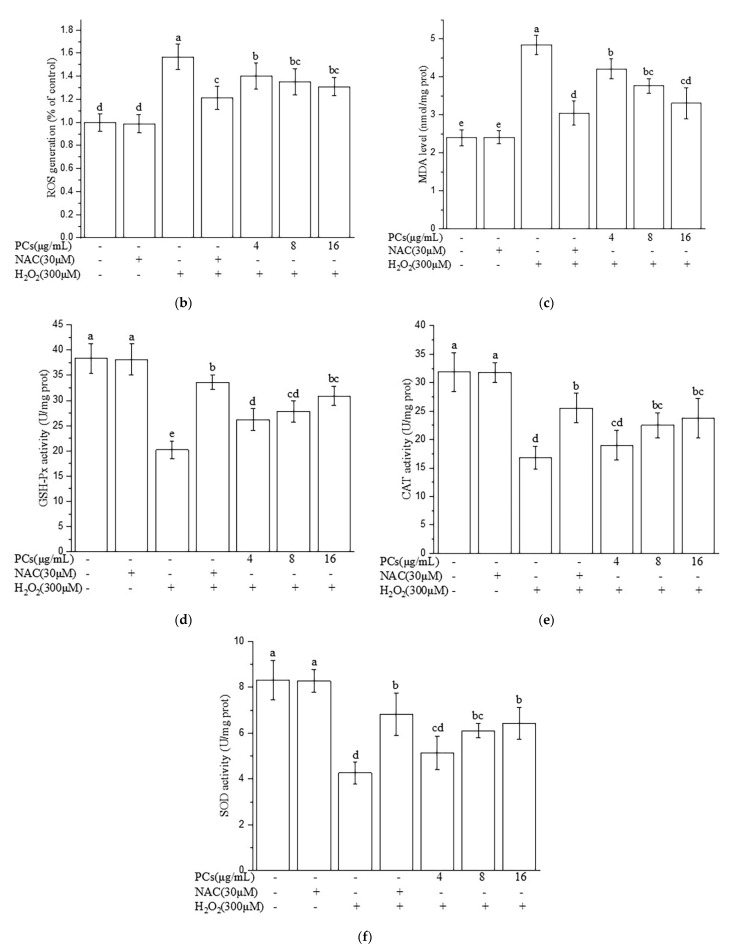
Effects of PCs on oxidative stress in H_2_O_2_-treated zebrafish larvae. (**a**) Representative fluorescence photomicrographs of zebrafish larvae; (**b**) ROS levels were measured via imaging software; (**c**) MDA levels; (**d**) GSH-Px activity; (**e**) CAT activity; (**f**) SOD activity. Data are expressed as the mean ± SD. All experiments were conducted three times. Values with different letters above are significantly different (*p* < 0.05, one-way ANOVA).

Treatment of PCs (4, 8, and 16 μg/mL) to zebrafish larvae reduced H_2_O_2_-induced increases in intracellular ROS formation (Figure 6a,b, *p* < 0.05 versus H_2_O_2_-treated group). Lipid peroxidation assays also indicated that PCs (4, 8, and 16 μg/mL) dose-dependently blocked H_2_O_2_-induced increases in MDA levels in zebrafish larvae (Figure 6c, *p* < 0.05 versus H_2_O_2_-induced group). Furthermore, GSH-Px activity was reversed by PCs (4, 8, and 16 μg/mL) treatment (Figure 6d, *p* < 0.05 versus H_2_O_2_-treated group). Additionally, PCs (8 and 16 μg/mL) increased H_2_O_2_-induced reductions in CAT and SOD activities (Figure 6e,f, *p* < 0.05 versus H_2_O_2_-induced group).

### 2.7. Effects of PCs on Nrf2/ARE Pathways in H_2_O_2_-Treated Zebrafish Larvae

**Figure 7:**
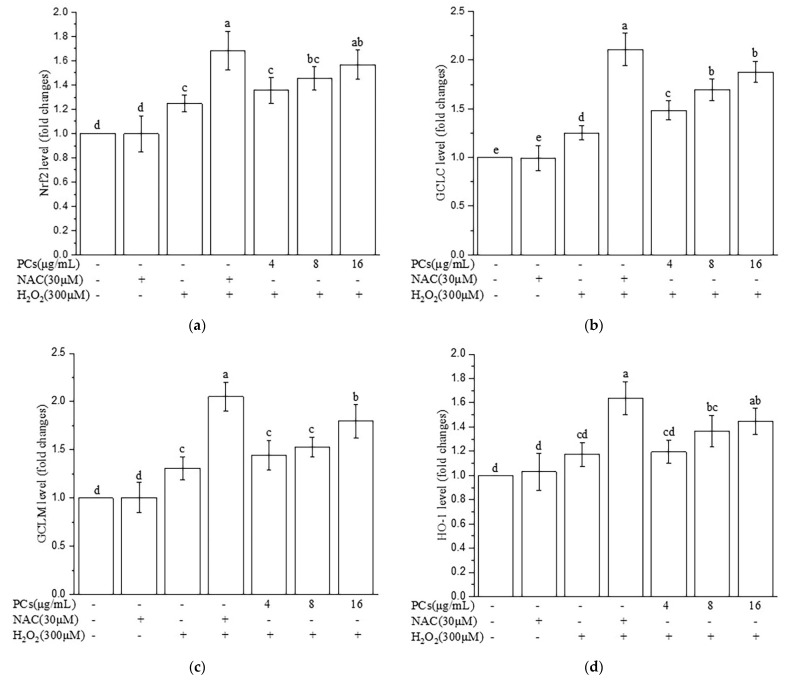

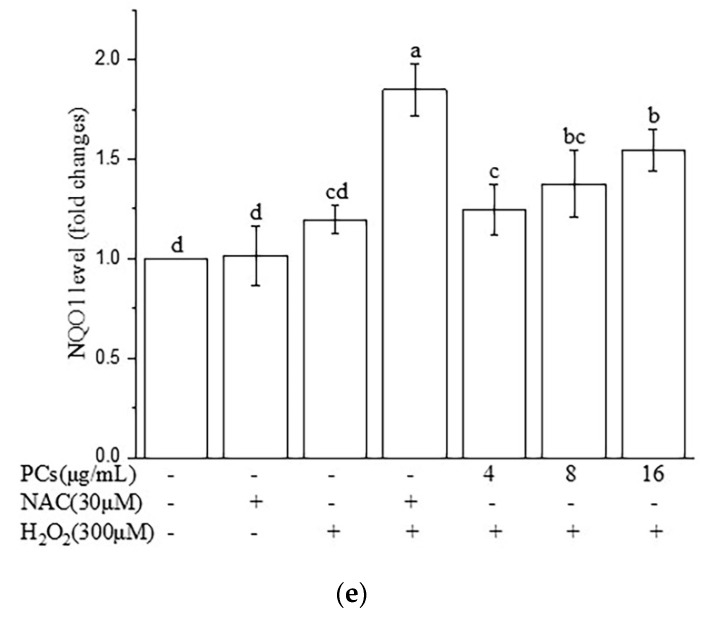
Effects of PCs on the Nrf2/ARE pathway in H_2_O_2_-treated zebrafish larvae. (**a**) Nrf2 levels; (**b**) GCLC levels; (**c**) GCLM levels; (**d**) HO-1 levels; (**e**) NQO1 levels. Data are expressed as the mean ± SD. Values with different letters above are significantly different (*p* < 0.05, one-way ANOVA test).

The protective effects of PCs treatment in H_2_O_2_-treated zebrafish larvae suggested that Nrf2/ARE pathways may have been involved. The expression levels of Nrf2, HO-1, NQO1, GCLC, and GCLM (Figure 7a–e, *p* < 0.05 versus H_2_O_2_-induced group) were up-regulated following PCs (16 μg/mL) treatment compared to levels after H_2_O_2_ treatment alone.

## 3. Discussion

Numerous studies have shown that PCs exhibit strong radical-scavenging and antioxidant activities [25,26,27], and that oxidative stress caused via the excessive production of ROS and/or impairment of the antioxidant defense system plays a critical role in neurodegenerative diseases [4,5,6,28,29]. However, the neuroprotective effects and mechanisms of PCs regarding oxidative damage are still unclear. Therefore, this study researched the effects and mechanisms of PCs in neuroprotection.

In the present study, the appropriate concentrations of PCs improved the levels of antioxidant enzymes, including GSH-Px, CAT, and SOD, but decreased the levels of ROS and MDA in models of oxidative damage compared with measurements using H_2_O_2_ alone. These present findings were consistent with previous studies which suggested that a low PCs supplement via diet can improve the activities of GSH-Px, CAT, and SOD in weaned piglets [30]. Another study also suggested that PCs significantly increased CAT and SOD activities and decreased MDA content, thus improving the quality of goat sperm [31].

The Nrf2/ARE pathway is a significant antioxidant pathway [32,33,34]. Normally, Nrf2 resides in the cytoplasm, where it is bound to the inhibitory protein, Keap1 [35,36]. Upon H_2_O_2_ stimulation, Nrf2 dissociates from Keap1, initiates the endogenous antioxidant defense system, and subsequently translocates into the nucleus [34,37,38]. It then interacts with ARE to activate a series of cell-protective and antioxidant genes, including GCLC, GCLM, NQO1, and HO-1 [39,40]. In response to oxidative stress, similar to changes seen during H_2_O_2_ stimulation, Nrf2 dissociates from Keap1 in the cytosol and is then translocated into the nucleus, binding to the ARE sequence to activate transcription of cryoprotective genes [41,42]. We observed that the appropriate concentrations of PCs facilitated nuclear localization of Nrf2 under H_2_O_2_-lesioning conditions in PC12 cells. Moreover, the appropriate concentrations of PCs markedly up-regulated the expression levels of NQO1, HO-1, GCLM, and GCLC, all of which can directly act to eliminate free radicals and oxidative molecules [43]. Hence, these results showed that the appropriate concentrations of PCs increased levels of the Nrf2/ARE pathway. Next, Nfr2 gene silencing via Nrf2 siRNA was used to further assess the role of Nrf2/ARE activation in PCs-mediated neuroprotection against H_2_O_2_-induced oxidative damage. Nrf2-siRNA-transfected cells indicated a marked decrease in Nrf2 expression. Simultaneously, we found that Nrf2 knockout abolished PCs-mediated protection against H_2_O_2_-treated impairments in cell viability, and Nrf2 knockout also abolished the antioxidant effects of PCs. These findings were accordant with previous study which suggested that improving activation of the Nrf2/ARE pathway contributes to neuroprotection [44]. Therefore, the present results indicate that the Nrf2/ARE pathway may represent a pharmacological target of PCs for the prevention of neurodegeneration.

## 4. Materials and Methods

### 4.1. Chemicals and Reagents

H_2_O_2_ was purchased from HUSHI (HUSHI, Shanghai, China). CCK-8, NAC were purchased from Beyotime (Beyotime, Shanghai, China). PCs, 2′,7′-dichlorofluorescin diacetate, MDA, GSH-Px, SOD diagnostic kits, and CAT diagnostic kits were purchased from Solarbio (Solarbio, Beijing, China). RNAiso Plus, PrimeScript RT reagent Kit with gDNA Eraser, and SYBR Premix Ex Taq II were purchased from Takara (Takara, Shiga, Japan). Nrf2-siRNA, control-siRNA, and lipofectamine 2000 were obtained from GenePharma (GenePharma, Shanghai, China). The following primary antibodies and corresponding secondary antibodies were obtained from Proteintech (Proteintech, Wuhan, China): Nrf2, HO-1, GCLC, GCLM, NAD(P)H: NQO1, lamin B, and GAPDH.

### 4.2. Cell Culture

PC12 cells were obtained from the National Collection of Authenticated Cell Cultures. The cells were maintained in DMEM medium supplemented with 10% fetal bovine serum and penicillin-streptomycin (100 U/mL; 100 μg/mL) in a humidified incubator at 37 °C with 5% CO_2_.

### 4.3. Cell Viability Assays

PC12 cells were seeded into 96-well plates at a density of 1 × 10^4^ cells/well. The cells were exposed to different concentrations of PCs or NAC (20 μM) for 24 h, after which they were incubated with 200 μM of H_2_O_2_ for 24 h. Next, 10 μL of CCK-8 solution was added into each well and incubated for 1 h, after which the absorbance was measured at 450 nm.

### 4.4. Fish Maintenance

Ethical approval for animal use was granted by the Animal Conservation and Use Committee of the Experimental Animal Center at Zhejiang University (ZJU20200125). Adult zebrafish (*Danio rerio*, AB strain) were obtained from the laboratory animal center of Zhejiang University and maintained at 28 ± 1 °C under a 14/10-h light/dark cycle [21]. The fish were fed *Artemia nauplii* twice daily. To produce embryos, adult zebrafish were placed overnight in breeding tanks at a 1:1, male:female ratio. Spawning was triggered within 2 h after the lights were turned on the next morning. Embryos were raised in embryo water at 28 °C [45].

### 4.5. ROS Measurements

To measure ROS production in vivo, zebrafish larvae (AB strain), 3 days post-fertilization (dpf) were exposed to NAC (30 μM) or various concentrations of PCs in the absence or presence of 300 μM of H_2_O_2_ for 4 days. Subsequently, at 7 dpf, the different treatment groups were transferred to a 24-well plate (10 zebrafish larvae per group) treated with 20 μM of 2′,7′-dichlorofluorescin diacetate solution, and were incubated for 60 min in the dark at 28.5 °C [46]. Next, the larvae were washed three times with embryo medium to remove excess 2′,7′-dichlorofluorescin diacetate. The stained larvae were then observed and imaged under an Olympus laser-scanning confocal microscope. The fluorescent intensity of each individual larva was quantified via ImageJ software.

To measure ROS production In vitro, PC12 cells were seeded into 6-well plates at a density of 2 × 10^4^ cells/well. The cells were exposed to different concentrations of PCs or NAC (20 μM) for 24 h and were then incubated with 200 μM of H_2_O_2_ for 24 h. The cells were incubated with 10 μM of 2′,7′-dichlorofluorescin diacetate solution in the dark for 30 min; thereafter, the dye solution was removed, and the cells were washed three times in phosphate-buffered saline. The cells were then observed and imaged under an Olympus laser-scanning confocal microscope. The fluorescent intensities of the cells were quantified via ImageJ software.

### 4.6. Assessment of MDA, GSH-Px, SOD, and CAT

Zebrafish larvae (AB strain) at 3 dpf were exposed to NAC (30 μM) or various concentrations of PCs in the absence or presence of 300 μM H_2_O_2_ for 4 days. The 7 dpf zebrafish were anesthetized with tetracaine and homogenized after euthanasia on ice. MDA, GSH-Px, CAT, and SOD were analyzed simultaneously, according to the manufacturer’s instructions. Each experiment contained 20 larvae, and each measured index was repeated in triplicate.

PC12 cells were seeded into 6-well plates at a density of 2 × 10^4^ cells/well. The cells were exposed to different concentrations of PCs or NAC (20 μM) for 24 h and were then incubated with 200 μM of H_2_O_2_ for 24 h. PC12 cells were next collected and incubated with lysis buffer. MDA, GSH-Px, SOD, and CAT activities were measured using corresponding assay kits, according to each manufacturer’s protocols. Each measured index was repeated in triplicates.

### 4.7. Total RNA Extraction, Reverse Transcription, and Quantitative Real-Time Polymerase Chain Reaction

Zebrafish larvae (AB strain) at 3 dpf were exposed to NAC (30 μM) or various concentrations of PCs in the absence or presence of 300 μM of H_2_O_2_ for 4 days. After treatments, RNA was extracted using RNAiso, following the manufacturer’s instructions. RNA was reverse transcribed using a PrimeScript RT Kit with gDNA eraser, following the manufacturer’s instructions (Takara, Shiga, Japan). Quantitative real-time PCR was performed with an Applied Biosystems Vii-7 Real-Time PCR system using SYBR Premix Ex Taq II. β-actin was used as a reference gene, and relative gene expression was calculated using the 2^−ΔΔCt^ method. The primer sequences utilized are listed in Table 1.

### 4.8. Nrf2 siRNA Transfection

Nrf2-siRNA was used to knockdown Nrf2. PC12 cells were seeded in 6-well culture plates and were then transfected with Nrf2-siRNA (80 nM) or control-siRNA using Lipofectamine 2000, according to the manufacturer’s instructions.

### 4.9. Preparation of Whole Cell, Cytoplasmic, and Nuclear Protein

For whole cell protein extraction, cells were collected and incubated with RIPA lysis buffer containing 1% PMSF and 1% protease inhibitor cocktail for 30 min on ice. Cell lysates were centrifuged, and the supernatant was collected and stored. For subcellular fractionation preparation, cell samples were processed using the nuclear and cytoplasmic protein extraction kit. The protein content was assayed using the BCA assay.

### 4.10. Western Blotting

Aliquoted protein samples were resolved by SDS-PAGE and transferred to polyvinylidene-difluoride membranes. The blots were then incubated with the following primary antibodies: Nrf2, Keap1, HO-1, NQO1, GCLC, GCLM, Lamin B, or GAPDH. The membrane was then washed with TBST for 5 min; this was repeated 5 times. Thereafter, blots were incubated in peroxidase-conjugated secondary antibodies. Finally, protein bands were visualized using an ECL-plus Western blotting detection reagent.

### 4.11. Locomotor Behavioral Assay

Zebrafish larvae (AB strain) at 3 dpf were exposed to NAC (30 μM) or various concentrations of PCs in the absence or presence of 300 μM of H_2_O_2_ for 4 days. Thereafter, at 7 dpf, these treated larvae were placed into 24-well plates (one fish per well and 12 larvae per group). We used Any-maze 4.73 to measure the locomotor behavior of zebrafish larvae, and calculated the total distance traveled in 10 min [47].

### 4.12. Statistical Analysis

Data are shown as the mean ± standard deviation (SD) of at least three independent experiments. One-way analysis of variance (ANOVA) and Duncan’s multiple range tests were used to determine statistical significance, and differences were considered significant when *p* < 0.05. Statistical analyses were performed via SPSS, version 20.0.

## 5. Conclusions

In conclusion, the present findings indicate that PCs exert neuroprotective effects via activating the Nrf2/ARE pathway and its downstream detoxification enzymes and antioxidant enzymes. Taken together, PCs may have protective effect on neurodegenerative disorders.

## Figures and Tables

**Table 1:** Sequence of primers for quantitative real-time PCR.

Genes	Forward Primer	Reverse Primer
β-Actin	CACTGAGGCTCCCCTGAATC	GGGTCACACCATCACCAGAG
Nrf2	CTGCTGTCACTCCCAGAGTT	GCCGTAGTTTTGGGTTGGTG
HO-1	AAGAGCTGGACAGAAACGCA	AGAAGTGCTCCAAGTCCTGC
GCLC	CTCCTCACAGTCACGGCATT	TGAATGGAGACGGGGTGTTG
GCLM	AAGCCAGACACTGACACACC	ATCTGGAGGCATCACACAGC
NQO1	AAGCCTCTGTCCTTTGCTCC	TGCTGTGGTAATGCCGTAGG

## Data Availability

Data is contained within the article.

## Sample Availability

Samples of the compounds are not available from the authors.
